# Promoting activity, Independence and stability in early dementia (PrAISED): a, multisite, randomised controlled, feasibility trial

**DOI:** 10.1186/s12877-019-1379-5

**Published:** 2019-12-16

**Authors:** Sarah E. Goldberg, Veronika van der Wardt, Andy Brand, Clare Burgon, Rupinder Bajwa, Zoe Hoare, Pip L. Logan, Rowan H. Harwood, on behalf of the PrAISED Study Group, John Gladman, Rhiannon Tudor Edwards, Tash Masud, Kavita Vedhara, Kristian Pollock, Vicky Booth, Roshan das Nair, Fiona Kearney, Martin Orrell, Vicky Hood, Kate Robertson, Juliette Lock, Claudio Di Lorito, Martyn Harling, Carys Jones, Melanie Heeley

**Affiliations:** 1 School of Health Sciences, University of Nottingham, Queens Medical Centre, Nottingham, NG7 2UH UK; 2 Division of Rehabilitation, Ageing and Wellbeing, University of Nottingham, Nottingham, UK; 3 Department of Primary Care, Rehabilitative and Preventative Medicine, Philipps University of Marburg, Marburg, Germany; 4 NWORTH, School of Health Sciences, Bangor University, Bangor, UK; 5 Nottingham University Hospitals NHS Trust, Nottingham, UK

**Keywords:** Dementia, Therapeutic exercise, Activities of daily living, Randomised controlled trial, Occupational therapy, Physiotherapy, Falls

## Abstract

**Background:**

We tested the feasibility of delivering and evaluating a complex therapy intervention which aimed to promote activity and independence for people with early dementia (PrAISED). Feasibility questions were on: recruitment, randomisation, intervention delivery, adherence and withdrawals, level of supervision required, adverse events, data collection and sample size assumptions.

**Methods:**

We conducted a three-arm, multi-site, single-blind, randomised controlled feasibility trial. Eligibility criteria were aged 65 years or older, diagnosed mild dementia or mild cognitive impairment, able to walk without human help, and communicate in English, no co-morbidities that prevented participation in cognitive assessment and capacity to give consent. Participants were recruited from Memory Assessment Service clinics and the ‘Join Dementia Research’ register.

Patient participants were randomised 1:1:1 to a high intensity supervision PrAISED intervention, moderate intensity supervision PrAISED intervention or brief falls prevention assessment and advice (control). The PrAISED intervention aimed for participants to complete three hours of PrAISED exercises a week for 12 months. It included individualised activity and exercise plans and supervised exercises with regular re-assessment and progression, and was delivered by occupational therapists, physiotherapists and rehabilitation support workers. Primary efficacy outcome was the Disability Assessment for Dementia (DAD), measured after 12 months. Secondary outcomes included physical activity, quality of life, mood, cognition, strength, balance, rate of falls, frailty and carer strain. Falls and activity were ascertained by monthly diary.

**Results:**

Between September 2016 and March 2017 we recruited 60 patient participants and 54 carer participants from two sites. Forty-nine patient participants completed a follow-up interview. Feasibility outcomes were mostly satisfactory, including recruitment and retention, intervention delivery and data completeness for most scales used. We could not maintain blinding of researchers at follow-up and experienced difficulties collecting data using some questionnaires and devices. Participants only completed a mean 77 (moderate supervision) and 71 (high supervision) minutes per week of PrAISED exercises over 12 months. We recorded 19 adverse events, none serious and related to the intervention.

**Conclusion:**

We conclude that with some adjustments to the trial protocol, it is feasible to deliver the PrAISED intervention and conduct a trial.

**Trial registration:**

ClinicalTrials.gov: NCT02874300 (first posted 22nd August 2016), ISRCTN: 10550694 (date assigned 31st August 2016).

## Background

Dementia comprises a group of progressive neurological diseases that affect memory and other cognitive functions, and which are severe enough to restrict daily function. Mild Cognitive Impairment (MCI) is a potential precursor state, with measurable cognitive deficits, but preserved functional ability, which progresses to dementia in over half of cases [1]. Neuropsychological deficits and falls risks are similar in people living with both diagnoses [2]. Remaining independent is a priority for people living with dementia and their carers [3, 4]. Ability to perform activities of daily living (ADL) deteriorates as the disease progresses. This is partly due to worsening cognitive impairment, including memory, apraxia, agnosia and executive function, but other aspects also contribute, including deconditioning, co-morbidities or injuries, failure to compensate or adapt to cognitive loss, and over-protectiveness or restriction of opportunity by carers, often in the name of safety. People living with dementia are especially prone to falls, physical illness, delirium, other crises and deterioration whilst in hospital.

A multi-disciplinary rehabilitation approach can address some of the problems. Physiotherapy aims to support activity through increases in strength and balance, improving gait, fitness, and confidence and reducing the risk of falls. Occupational therapy can support independence through cognitive rehabilitation and risk enablement approaches. Health psychology can support the individual to make changes to their daily life to become more active by linking these desired changes to basic psychological needs, and addressing barriers.

A Cochrane systematic review [5] reported evidence that exercise programs for people with dementia improved ADL, but the quality of evidence was rated as poor. High intensity home-based exercise reduced rate of loss of ADLs and falls [6], and functionally-directed home-based therapy can also increase activity [7], although this has not been replicated [8]. Many other trials of simple interventions in dementia have failed to improve outcomes; for example, a moderate intensity group exercise programme failed to improve cognition or ADL [9]. The optimum ‘dose’ of exercise required to improve ADLs in people with dementia is not clear; most studies aimed for one hour of exercise three times a week for 24 weeks [5], which is similar to the amount of exercise required to reduce risk of falls. The World Health Organisation (WHO) recommend that people aged 65 years and older with poor mobility should do balance challenging exercise on 3 or more days a week and muscle strengthening activities on two more days [10]. Inconsistency in trial results may be due to variable or low intervention adherence, ranging from 16 to 100% in a review [unpublished observation from Di Lorito, C. Bosco, A. Booth, V. Goldberg, S. Harwood R.H. and van der Wardt, V.].

We systematically developed a complex intervention which aims to keep people living with early dementia or mild cognitive impairment independent and active, whilst reducing their risk of falls, and focusing on promoting uptake and adherence [11]. The intervention was developed over five years [4, 11, 12], by a team including occupational- and physio-therapists, health psychologists, nurses, geriatricians and carer representatives.

Feasibility trials are increasingly used to test trial methods, the practicality of delivering the intervention, and parameters needed to plan recruitment and estimate sample size, in advance of conducting a fully-powered randomised controlled trial. Methods for designing, and interpreting feasibility studies and responding to their findings have been published [13–15].

## Methods

### Study design

We undertook a pragmatic, parallel group, single-blinded, randomised controlled feasibility trial at two study sites, recruiting from Memory Assessment Services (dementia diagnostic services) and the Join Dementia Research register, a United Kingdom National Institute for Health Research (NIHR) web-based initiative to increase participation in dementia research [16]. The full rationale and protocol have been published [17]. There were no significant changes to the research plan during the feasibility study. The CONSORT 2010 checklist of information to include when reporting a pilot or feasibility trial is available in Additional file 1.

### Participants

Patient participants were identified by clinicians working in Memory Assessment Services and referred to the research team if interested, or by researchers through the Join Dementia Research Register. We included patient participants who were age 65 years or over and had diagnosed mild dementia or mild cognitive impairment (Montreal Cognitive Assessment (MoCA) score of 15–25 [18]; Mini Mental State Examination (MMSE) of 18–26 [19] or Addenbrookes Cognitive Examination III (ACEIII) 60–94 [20], depending on what assessments clinics used). Patient participants had to be able to walk without human assistance and communicate in English, have no co-morbidities that prevented participation in cognitive assessment and capacity to give informed consent (formally assessed by a researcher). A family member or informal carer of the patient participant who could communicate in English was recruited, if willing. Written consent was taken from both patient and carer participants. We chose to study people with mild impairments as our intervention was designed to prevent or slow decline amongst those still well enough to engage and learn new lifestyle activities.

Researchers and NIHR Clinical Research Network clinical support officers (CSO) recruiting patient participants and collecting data had two days training in study procedures. Two researchers or CSOs visited the patient and carer participants in their own home to complete a screening questionnaire, discuss the study and take written informed consent. The commitment needed to complete the study (if randomised to one of the active arms) was emphasised to avoid later withdrawals. Baseline data were collected from the patient and carer participant. Some patient participant data were collected from the carer participant to reduce burden. Visits with two researchers or CSOs allowed the patient and carer participants to be interviewed concurrently in different rooms, reducing the overall time of the interview and allowing the participants to respond freely.

To describe the patient participants, we collected demographic data, past medical history, medications, and falls risk factors from the carer participant and measured cognition by completing a standardised mini mental state examination (sMMSE) [19] with the patient participant.

### The intervention

We tested two versions of the PrAISED intervention, which differed in the amount of professional supervision offered. Both versions aimed for the patient participant to complete three hours of PrAISED exercises each week for the 12 month intervention period. Both versions included assessment, creation of an individualised tailored exercise and activity plan, supervised exercises and activity, and regular reassessments and progression. Patient participants in the high intensity supervision group received up to 50 visits from a therapist or rehabilitation support worker (RSW) over a period of one year. Patient participants in the moderate intensity supervision group received 9 visits from a therapist (or two therapists for initial assessment visits) and three phone calls over a three month period, and encouragement to continue the programme after professional supervision ceased.

The PrAISED physical exercises included balance challenging, strength building, dual-task training and gait re-education. Some exercises could be gained through functional strategies which aimed to maintain or improve independence. Patient participants in the high intensity supervision group received visits that were tapered over the year, starting at twice weekly for three months, then weekly for three months, bi-monthly for three months and monthly for the last three months. Patient participants in the moderate intensity supervision group received tapered visits in the first three months of the twelve month intervention period. The supervision regimens were based on precedents from the literature [6, 21]. Both interventions included motivational strategies to encourage adherence to, and persistence with, the planned exercises and activities. Participants were encouraged to continue their exercise and activities beyond the supervised period and were provided information on appropriate community exercise groups in their area. Therapists and RSWs delivering the intervention received three days training prior to the start of the study and 2 days training during the intervention delivery period. The control group had a single falls prevention assessment and advice with one to two follow-up visits by a therapist if indicated. Standard care controls are common practice in activity and exercise interventions for people with dementia [6, 9, 21].

### Objectives

A feasibility study aims to answer the question “can this study be done?” [22]. They play an important role in the subsequent design of a fully powered trial [13]. Specific trial feasibility questions were [17]:

i) Can we recruit and randomise patient participants at a sufficient rate (set at > 2.5 participants per week from 2 sites)?
ii) Can we deliver the intervention across sites and in patient participants’ homes?
iii) Is the intervention adhered to and how many withdraw (Set at > 75% of participants retained in the study)?
iv) What level of supervision intensity is required that will enable engagement at a level likely to be effective?
v) Are there any unexpected or adverse consequences?
vi) Can we collect blinded trial data at baseline and follow- up without burdening participants (set at < 20% missing primary outcome)?
vii) Are our sample size assumptions correct?

### Health status outcomes

Outcomes were measured at baseline and 12 month follow-up visits.

Activities of daily living were measured using the Disability Assessment for Dementia scale (DAD) [23], completed by the carer participant and the Nottingham Extended Activities of Daily Living Scale (NEADL) [24], completed by patient participant.

Cognition was measured using Cambridge Neuropsychological Test Automated Battery (CANTAB) tests [25, 26]: Paired Associated Learning (PAL), Attention Switching Task (AST), Spatial Working Memory (SWM). We also measured a verbal fluency test (from MoCA) [18]. We used these tests as previous studies had shown that it was unlikely that exercise would improve global cognition scores, but may influence specific elements such as executive function. The clinical dementia rating [27] was completed by the researcher.

Sitting and standing blood pressure and pulse rate were measured using an automated sphygmomanometer (Omron, Milton Keynes, UK). The Berg Balance Scale [28], leg and hand strength (Lafayette dynamometer, Loughborough, UK), timed up and go (TUG) test and dual task TUG (whilst counting backwards in 3’s) were measured directly with the patient participant. The SHARE Frailty instrument [29], and International Physical Activity Questionnaire (IPAQ) [30] were completed by carer participant.

Quality of life was measured from the patient participant using EQ-5D-3L [31]; and Dementia Quality of Life Scale (Demqol) [32]. Demqol proxy was completed by the carer participant. Anxiety and depression (Hospital Anxiety and Depression Scale, HADS) [33] and Falls Efficacy Scale – International (FES-I) [34] were completed by the patient participant. Carer participants completed the carer strain index (CSI) [35].

Throughout the year of follow-up, patient participants were asked to complete and return monthly falls and exercise calendars, by post, in a stamped addressed envelope provided by the researchers or CSOs, prompted by monthly telephone reminders. At baseline, six and 12 months patient participants were asked to wear and then return, by post, a tri-axial accelerometer for one week to objectively measure activity. A range of accelerometers were provided to participants. See Table 1 for data collection time points.

**Table 1:** Data collection time points

Scale or measure	Baseline	6 months	12 months	Completed by
Disability in dementia scale (DAD)	X		X	Carer participant
Nottingham Extended Activities of Daily Living Scale (NEADL)	X		X	Patient participant
Demographics	X			Carer participant
Past Medical History	X			Carer participant
Medications	X			Carer participant
Falls risk factors	X			Carer participant
Standardised Mini Mental State Examination (sMMSE)	X			Patient participant
Verbal fluency	X		X	Patient participant
Cambridge Neuropsychological Test Automated Battery (CANTAB) tests	X		X	Patient participant
Blood pressure and pulse	X		X	Patient participant
Berg Balance scale	X		X	Patient participant
Leg and hand strength	X		X	Patient participant
Timed up and go test and dual task timed up and go test.	X		X	Patient participant
SHARE frailty instrument	X		X	Patient participant
International Physcial Activity Questionnaire (IPAQ)	X		X	Carer participant
EQ-5D-3L	X		X	Patient participant
Dementia Quality of Life Scale (Demqol)	X		X	Patient participant
Hospital Anxiety and Depression Scale (HADS)	X		X	Patient participant
Falls Efficacy Scale – International (FES-I)	X		X	Patient participant
Carer strain index	X		X	Carer participant
Steps (accelerometer)	X	X	X	Patient participant

Adverse events were monitored and reported to the principal investigator at each site by therapists, RSWs and researchers during telephone contacts to prompt diary return. Adverse events were defined as hospitalisation, or incidents which were life threatening, causing persistent or significant disability or incapacity, or an incident, injury or symptom related to therapy sessions or exercise undertaken independently.

### Randomisation

Participants were randomly allocated to one of the three arms, stratified by site, presence of a co-resident carer and history of previous falls (to control for confounding variables) using a dynamic adaptive allocation algorithm [36] accessed by a secure web portal held at the NWORTH clinical trials unit (CTU), Bangor University. The randomisation system was maintained by a statistician independent of the analysis and research teams to ensure blinding of analysis. The researchers and CSOs entered the patient participant’s details into the web portal. Notification of allocation was sent to ‘unblinded’ members of the research team who notified the therapists of allocation. The therapists then informed the patient participant. Patient participants were randomised 1:1:1 to high intensity supervision, moderate intensity supervision or standard falls prevention assessment and advice (the control arm).

### Concealment

We could not blind the patient and carer participants to the intervention. We took measures to ensure the researchers collecting primary outcome data were blind to allocation. For example, the ‘blinded’ researchers sat in a different office, spreadsheets identifying allocation were password protected to avoid accidentally seeing allocations and we took care in meetings not to reveal allocation. Randomisation emails giving details on allocation were not sent to ‘blinded’ researchers.

### Data management

Data were entered into a MACRO database. Data which could ‘unblind’ researchers was kept separately from ‘blinded’ data on MACRO with access given as appropriate. Database management and data cleaning were managed by the CTU. Source data verification was undertaken for 5% of data from both carers and patient participants at baseline and follow-up at each site. Source data to be verified were randomly selected by the CTU and researchers completed the verification checks.

### Sample size calculation

A sample size of 60 was chosen to be sufficient to answer feasibility questions. A sample of this size would give adequate precision using a confidence interval approach [37] which considers the likelihood of a future definitive study finding a relevant effect size.

### Statistical analysis

A statistical analysis plan was drawn up by the trial statistician prior to completion of recruitment. Statistical analyses provided descriptive statistics on recruitment and retention rates, participation, missing data, adverse events, follow-up, and falls ascertainment rates, and the distributions of key variables required for main trial sample size calculation. Mean health status variables were calculated by group, at baseline and follow-up, with mean differences. Standardised effect size estimates from analyses of covariance (ANCOVA), equivalent to Cohen’s d [38], were calculated. A Cohen’s d of 0.2 to 0.5 is a small effect size; 0.5 to 0.8 is a medium effect size and greater than 0.8 is a large effect size [38].

### Progression to trial

We set discontinuation rules of recruitment (> 2.5 participants per week), retention (> 77%), intervention set up at sites, data collection (< 20% missing primary outcome).

## Results

### Recruitment and randomisation rate

**Fig. 1:**
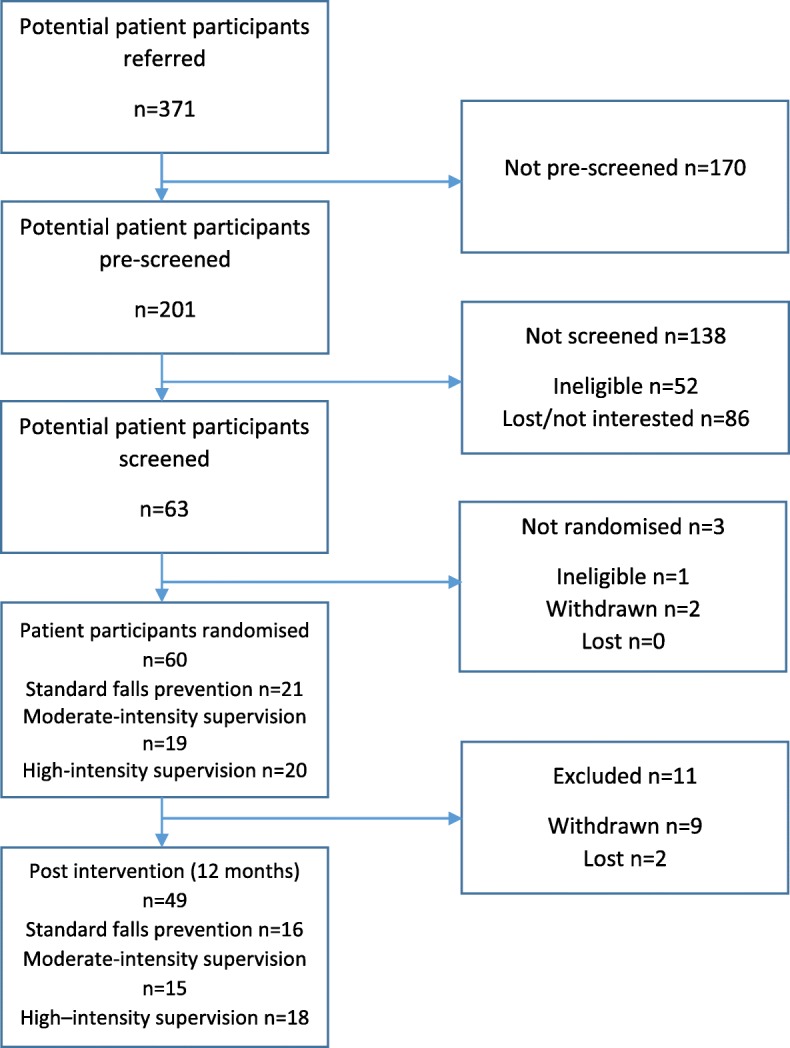
Consort diagram

Between September 2016 and March 2017 371 people with mild dementia or MCI were referred to the study and 201 were pre-screened. Of these, 52 were ineligible and 86 either not interested or lost. We screened the remaining 63, one was ineligible and two withdrew. We recruited 60 patient participants and 54 carer participants, on schedule (consort diagram, Fig. 1). Seventeen were recruited via Join Dementia Research. Patient participants had a mean age of 76 years (range 65–91), 34/60 (57%) were male and 45/60 (75%) married. Most were white ethnicity (58/60, 97%); lived with another person (48/60, 80%); had received secondary education 32/60 (53%) and many had received further education 26/60 (43%). Mean sMMSE was 25.6/30 (range 18–30), 55/59 (93%) had a diagnosis of dementia. Carer participants were younger with a mean age of 68 years (range 33–87), 45/60 (75%) were spouses and 39/60, (65%) were female (Table 2). Twenty one patient participants were randomised to standard care, nineteen to the moderate intensity supervision intervention and twenty to the high intensity supervision intervention. There was a gender imbalance between groups with 62% of the standard care group being female compared to 37 and 30% in the moderate and high intensity supervision groups; groups were otherwise well-matched at baseline.

**Table 2:** Distribution of participant categorical demographic variables split by the allocation group

	Standard falls prevention *N* = 21	Moderate-intensity supervision *N* = 19	High-intensity supervision *N* = 20	Total n/N (%)
Patient Participants				
Gender Female n (%)	13 (62%)	7 (37%)	6 (30%)	26/60 (43%)
Marital Status Married n (%)	16 (76%)	14 (74%)	15 (75%)	45/60 (75%)
Ethnic Group White n (%)	20 (95%)	18 (95%)	20 (100%)	58/60 (97%)
First Language English n (%)	20 (95%)	19 (100%)	20 (100%)	59/60 (98%)
Living Alone n (%)	5 (24%)	4 (21%)	3 (15%)	12/60 (20%)
sMMSE mean/30 (sd)	25.9 (2.4)	24.8 (3.6)	26.2 (3.2)	25.6 (3.1)
Diagnosed dementia	19/20 (95%)	18/19 (95%)	18/20 (90%)	55/59 (93%)
Education				
Primary school education or less	0 (0%)	1 (5%)	0 (0%)	1/60 (2%)
Secondary education	11 (52%)	11 (58%)	10 (50%)	32/60 (53%)
Further education	9 (43%)	7 (37%)	10 (50%)	26/60 (43%)
Not Answered	1 (5%)	0 (0%)	0 (0%)	1/60 (2%)
CARER PARTICIPANTS				
Carer Relationship with Person in Study				
Husband/wife/partner	15 (71%)	15 (79%)	15 (75%)	45/60 (75%)
Son/daughter	3 (14%)	3 (16%)	3 (15%)	9/60 (15%)
No carer recruited	3 (14%)	1 (5%)	2 (10%)	6/60 (10%)
Carer Gender Female	10 (48%)	13 (68%)	16 (80%)	39/60 (65%)

### Intervention delivery

The intervention was successfully delivered in participant’s homes, across the two sites with the control group receiving a mean of 2 sessions, the moderate intensity supervision group receiving 12/12 (100%) sessions (including telephone calls) and the high intensity supervision group receiving 33/50 (66%) sessions, although some sessions had more than one therapist attending.

### Adherence and withdrawal

Three hundred and ninety one out of five hundred and twenty eight (74%) of the exercise and falls calendars were returned. From these calendars, mean PrAISED related physical activity minutes per week for both intervention groups was 72 min (Standard deviation (SD) = 63) when calculated for the time between baseline and follow up (approximately 11 months). In the moderate intensity supervision group, participants completed a mean of 77 min (SD = 71; range 15 to 228 min) per week and 71 min (SD = 56; range 11 to 246 min) in the high intensity supervision group. Those in the control group had not been given PrAISED exercises to complete.

Seven participants withdrew from therapy before the 12 month outcome visit (three from the high intensity supervision group, two from the moderate intensity supervision group and two from the control group). Two participants gave depression as the reason for withdrawing, one was feeling overwhelmed, one felt he wasn’t eligible because he was physically well, one thought it was the wrong time and that the visits stopped her going out and doing what she wanted to do, one withdrew due to physical and mental deterioration and one withdrew due to bereavement and feeling the exercises were not appropriate to her age due to risk of falls.

### Data collection and blinding

Between September 2017 and March 2018, 49/60 (82%) participants completed the follow-up visit with nine withdrawals and two participants lost to follow-up. 16/21 (76%) participants from the control group completed the follow up visit, 15/19 (79%) from the moderate intensity supervision group and 18/20 (90%) from the high intensity supervision group. Assessment interviews proved lengthy with a median duration of 115 min. 32/52 (62%) patient participants reported that the time taken was acceptable 16/52 (31%) considering that it was too long. Missing data at baseline and follow-up varied across the different measures and questionnaires (Table 3). The primary outcome measure, the DAD, was completed for 52/60 (87%) participants at baseline and 46/49 (94%) participants at follow-up. DAD is an informant rated scale. Six participants were recruited without a carer, so did not provide DAD data. A ceiling effect was seen with 11/52 participants at baseline and 4/46 at follow-up gaining full scores on the DAD. CANTAB assessments and accelerometer data showed considerable missing data due to technical problems. The carer participants reported finding the IPAQ difficult to complete.

**Table 3:** Crude scores on oncome measures according to randomisation group for the PrAISED feasibility study

Measure	Group	N	Baseline Mean (SD)	Follow-up Mean (SD)	Difference	Interpretation
DAD Score/100	Control	14	76 (20)	58 (33)	−18	deterioration
	Moderate	15	83 (17)	66 (30)	−17	deterioration
	High	15	76 (25)	68 (25)	−8	deterioration
NEADL Score/22	Control	10	16 (4)	15 (6)	−1	deterioration
	Moderate	13	17 (5)	15 (5)	−1	deterioration
	High	17	16 (5)	15 (5)	−1	deterioration
DEMQoL /112	Control	13	85 (18)	86 (20)	1	improvement
	Moderate	14	88 (8)	91 (15)	3	improvement
	High	17	86 (16)	86 (16)	0	unchanged
DEMQoL Proxy/124	Control	15	91 (15)	92 (15)	1	improvement
	Moderate	15	98 (14)	101 (13)	3	improvement
	High	16	91 (15)	94 (18)	2	improvement
Berg Balance Scale/56	Control	11	49 (9)	43 (17)	−7	deterioration
	Moderate	13	52 (2)	52 (3)	−1	deterioration
	High	13	50 (10)	50 (6)	0	unchanged
Falls Efficacy Scale-International/64	Control	12	28 (11)	34 (15)	6	deterioration
	Moderate	13	25 (13)	23 (11)	−2	improvement
	High	13	23 (8)	25 (12)	2	deterioration
IPAQ Physical Activity Score*	Control	12	1456 (1466)	989 (1187)	− 467	deterioration
	Moderate	14	2489 (2464)	1323 (923)	− 1166	deterioration
	High	14	1484 (1658)	1729 (1873)	245	improvement
HADS Anxiety/21	Control	11	5.2 (3.7)	4.8 (4.9)	−0.4	improvement
	Moderate	13	6.5 (3.3)	4.5 (2.4)	−2.0	improvement
	High	14	6.2 (4.0)	7.0 (4.4)	0.8	deterioration
HADS Depression/21	Control	11	4.4 (3.7)	3.7 (3.0)	−0.6	improvement
	Moderate	11	4.0 (3.6)	3.3 (2.0)	−0.7	improvement
	High	15	4.8 (3.9)	4.7 (3.0)	−0.1	improvement
SHARE Frailty Index (no maximum score)	Control	14	1.5 (1.7)	1.9 (2.0)	0.4	deterioration
	Moderate	14	0.6 (1.6)	0.4 (1.4)	−0.2	improvement
	High	14	1.2 (1.6)	1.4 (2.0)	0.2	deterioration
Accelerometer - Total Number of Steps	Control	11	19,141 (13284)	23,713 (15088)	4572	Technical problems means not possible to interpret
	Moderate	13	15,947 (20503)	32,445 (26220)	16,498	
	High	11	14,496 (20785)	21,389 (17503)	6893	
Timed Up and Go (seconds)	Control	14	19 (10)	25 (18)	7	deterioration
	Moderate	13	12 (3)	13 (6)	1	deterioration
	High	15	16 (9)	15 (13)	−1	improvement
Dual Task Timed Up and Go (seconds)	Control	13	23 (10)	28 (17)	5	deterioration
	Moderate	10	20 (11)	27 (29)	7	deterioration
	High	11	17 (8)	15 (7)	−3	improvement
EQ-5D-3L Index	Control	13	0.8 (0.2)	0.7 (0.2)	0	unchanged
	Moderate	12	0.7 (0.3)	0.8 (0.3)	0.1	improvement
	High	16	0.8 (0.2)	0.8 (0.2)	0	unchanged

*score calculated as minutes of exercise multiplied by intensity, for each activity performed

Despite efforts to blind researchers collecting follow-up data the researchers were able to correctly identify allocation (intervention or control) for 42/49 (86%) outcome visits.

### Supervision intensity required to enable engagement

There was a trend towards better performance on the primary outcome measure, DAD, and less deterioration from baseline, with higher intensity supervision. Mean DAD scores were 76/100 at baseline versus 58/100 at follow-up for the control group, 83 versus 66 for the moderate intensity supervision group and 76 versus 68 for the high intensity group. Cohen’s d was 0.03 (− 0.7 to 0.8) for moderate (no effect) and 0.43 (− 0.3 to 1.2) for high intensity supervision (a small effect size), compared with control (Tables 3 and 4) [38].

The Berg Balance Scale showed a large and statistically significant positive effect size for the moderate intensity d = 0.9, 95% CI [0.1, 1.8] and high intensity d = 1.0, 95% CI [0.1, 1.8] groups compared with control. Fear of falling improved for the moderate intensity supervision group d = − 0.8, 95% CI [− 1.7, − 0.03] (Table 4).

**Table 4:** Standardised effect sizes estimates for moderate and high intensity supervision intervention and missing data

Measures	Effect Size for Moderate Intensity (95% Confidence interval)*	Effect Size for High Intensity (95% Confidence interval)*	Missing Data N (%) at baseline from the 60 recruited participants	Missing Data N (%) at follow up from the 49 remaining participants
DAD Score	0.03 (−0.7 to 0.8)	0.43 (−0.3 to 1.2)	**8/60 (13%)**	**3/49 (6%)**
NEADL Score	0.2 (−0.7 to 1.0)	0.3 (− 0.5 to 1.1)	**3/60 (5%)**	**7/49 (14%)**
DEMQoL	0.2 (−0.6 to 1.0)	−0.1 (− 0.8 to 0.7)	**0/60 (0%)**	**5/49 (10%)**
DEMQoL Proxy	0.4 (−0.4 to 1.1)	0.1 (−0.6 to 0.8)	**7/60 (12%)**	**3/49 (6%)**
Berg Balance Scale	0.9 (0.1 to 1.8)	1.0 (0.1 to 1.8)	**7/60 (12%)**	**8/49 (16%)**
Falls Efficacy Scale-International	−0.8 (−1.7 to − 0.03)	−0.5 (−1.3 to 0.3)	**2/60 (3%)**	**9/49 (18%)**
IPAQ Physical Activity Score	0.2 (−0.6 to 1.0)	0.6 (−0.2 to 1.4)	**3/60 (5%)**	**7/49 (14%)**
HADS Anxiety	−0.5 (−1.3 to 0.4)	0.5 (− 0.3 to 1.3)	**4/60 (7%)**	**9/49 (18%)**
HADS Depression	−0.2 (−1.0 to 0.6)	0.3 (− 0.4 to 1.1)	**1/60 (2%)**	**12/49 (24%)**
SHARE Frailty Index	−0.7 (−1.4 to 0.1)	− 0.2 (− 0.9 to 0.5)	**4/60 (7%)**	**6/49 (12%)**
Pedometer-Total Number of Steps	0.4 (−0.4 to 1.2)	− 0.2 (− 1.0 to 0.7)	**12/60 (20%)**	**7/49 (14%)**
Verbal Fluency - Correct Words	0.5 (−0.3 to 1.2)	0.4 (− 0.3 to 1.1)	**1/60 (2%)**	**4/49 (8%)**
Timed Up and Go	−0.4 (−1.2 to 0.3)	− 0.75 (− 1.5 to 0.00)	**0/60 (0%)**	**7/49 (14%)**
Dual Task Timed Up and Go	0.3 (−0.5 to 1.2)	− 0.4 (− 1.2 to 0.4)	**5/60 (8%)**	**13/49 (27%)**
EQ-5D-3L Index	1.4 (0.5 to 2.2)	0.3 (−0.4 to 1.0)	**1/60 (2%)**	**8/49 (16%)**

Note: 0–0.3 = trivial; 0.3–0.5 = small; 0.5–0.8 = moderate; > 0.8 = large

*Positive values show an effect in favour of intervention group

### Unexpected or adverse outcomes

There were 19 recorded adverse events. Five were related to the intervention but not serious, 12 were serious but not related, 2 were neither serious nor related to the intervention. They were all recorded in the active intervention groups, but were subject to ascertainment bias as these groups had much more contact with therapists.

Only 12 falls were reported by participants over the 12 months, fewer than anticipated [2, 17]. The incident rate ratios (IRR) were 0.68, (95% CI 0.16, 2.8) and 1.20, (95% CI 0.32, 4.5) for moderate and high intensity supervision, neither being statistically significant.

### Sample size assumptions

For the main trial primary outcome is disability at 12 months. We calculated that a sample size of 184 participants per group, including 23% attrition (based on previous studies [6, 39], has 80% power to detect changes in the disability outcome, DAD, with effect size 0.5 (11 points on a baseline of 70, standard deviation 22, data from [39, 40]).

The results from this feasibility study broadly supported our original sample size calculation for the high intensity supervision group and we have not revised the original sample size calculation based on these results.

## Discussion

We tested the feasibility of evaluating a programme of therapeutic exercise and activity for people with mild dementia or mild cognitive impairment delivered over one year, with moderate and high levels of professional supervision. All criteria for progression to the fully powered main trial were met. We recruited appropriate participants at a sufficient rate, delivery of and adherence to the intervention, and retention in the study were satisfactory. Randomisation and data management systems worked well. There were no unexpected or adverse consequences of the intervention. Completion rates for baseline and outcome data were satisfactory for most but not all measures used, but required a carer as a reliable informant and blinding of researchers proved unfeasible. Asking the carer participant to complete some patient participant scales reduced burden on the patient participant. The intervention was feasible and safe to deliver. We found our sample size estimates for the main trial to be reasonable.

### Recruitment and intervention delivery

Our study was conducted at two sites with different ways of organising staff to deliver the intervention. The ‘Join Dementia Research’ register proved useful for recruitment, but may have skewed the population to more educated and younger participants.

### Adherence and withdrawal

The adherence rate for the supervised activity and exercise sessions of 66% for the high intensity supervision version and 100% for the moderate intensity supervision version is comparable to other studies of this kind which show a mean adherence rate of 71% [unpublished observation from Di Lorito C, Bosco A, Booth V, Goldberg S, Harwood RH, van der Wardt V]. However, mean activity per week was only 72 min of PrAISED exercises a week, falling considerably short of the required ‘dose’ of 180 min and WHO recommendations [10]. Therapist training has been changed for the main trial with a greater focus on motivation and adherence. Our attrition rate of 18% was acceptable compared to similar studies (23% for FiNALEX [6]; 9% for Wesson [21]).

### Supervision intensity required to enable engagement

The standardised effect size estimates for the primary and some intermediate outcome measures (whilst difficult to interpret due to small sample size) suggest the intervention, delivered with a high level of supervision, could feasibly show a positive effect at 12 month outcomes in a fully powered comparison (in other words, the intervention is not shown to be futile). The PrAISED intervention required patient participants to complete 3 h of PrAISED exercises a week for 12 months, but differed according to the amount of professional contact they received. Of the two intervention groups, the greater effect size was seen with the high intensity supervision group. Measured adherence was not different between these groups, but these data are difficult to collect, not least because the difference between an ‘exercise’ and an ‘activity’ can be difficult to determine, and therapists reported that some participants chose to do ‘activities’ over ‘exercises’ as time went by. Care may be enhanced in the therapy arms through the interactions between participant and therapist (such as if the therapist referred the patient participant to services for a problem not related to the intervention [41]). We cannot be sure whether it was the ‘social’ contact or the therapy programme that changed outcomes, however, in complex intervention trials all elements are typically considered together. It was not feasibly to have a sham control and it is common practice to have a ‘usual care’ control arm [6, 9, 21] for activity and exercise interventions for this patient group. Our research suggests participants are less likely to do the exercise without human contact (through supervision or support from carers) [42].

### Collection of blinded data

Unblinded trials can result in bias [43], however, we found blinding was impossible to maintain in practice. Issues of bias in outcome data collection need to be addressed through specific anti research bias training, which we have put in place for the main trial.

Many people with dementia live alone in the community and some do not have any family carers. We wanted to be inclusive in our recruitment, but found we could not complete primary outcome data with this policy and will restrict our inclusion criteria for the main trial to those with a carer willing to take part.

The DAD as a primary outcome measure has been used before in similar trials; is recommended [44] and was well completed when a carer was available. However, the DAD did show a ceiling effect at baseline and outcome; potentially reducing the chances of demonstrating effectiveness. Better ADL outcome measures for people with early dementia are needed. Measurement of physical activity using accelerometers was inaccurate due to technical difficulties, which were resolved during the feasibility trial and which has informed researcher training. We selected the best performing accelerometer for the main trial (Misfit Shine 2). Carer participants found the IPAQ difficult to complete and we have changed it to the Longitudinal Aging Study Amsterdam physical activity questionnaire for the main trial [45]. An independent CTU managed the data and undertook statistical analysis blind to intervention group. Some scales had high levels of missing data resulting in some being dropped or changed and instructions on completion being clarified for the planned main trial.

### Unexpected or adverse outcomes

There were no serious adverse events related to the intervention, suggesting the intervention does not cause harm. In a trial where one group has considerably more contact than the other, there is the problem of differential reporting of adverse events, and comparing rates of adverse events in different groups is impossible to interpret. We have embedded safety parameter variables (mortality, hospitalisation, falls, injury) in the outcome dataset, which is collected independently of contact with therapists delivering the clinical intervention. This approach was agreed by our Data Monitoring Committee.

### Sample size assumptions

Our sample size assumptions are shown to be reasonable.

## Conclusions

We have tested the feasibility of a multisite randomised controlled trial using a three arm study and found, that a larger trial is feasible with some adjustments to methods. The adjustments to the trial protocol identified in this study demonstrate the importance of conducting a feasibility study prior to full trial.

## Supplementary information

**Additional file 1.** CONSORT 2010 checklist of information to include when reporting a pilot or feasibility trial.

## Data Availability

The dataset analysed during this study are available from the corresponding author on reasonable request.

## Abbreviations

ACE: Addenbrooke’s Cognitive Examination
ADL: Activities of Daily Living
ANOVA: Analyses of Covariance
AST: Attention Switching Task
CANTAB: Cambridge Neuropsyhcological Test Automated Battery
CI: Confidence interval
CSI: Carer strain index
CSO: Clinical support officer
CTU: Clinical Trials Unit
DAD: Disability Assessment for Dementia
Demqol: Dementia Quality of Life Scale
EQ-5D-3L: Euroqol – five dimensions – 3 levels
FES-I: Falls Efficacy Scale – International
HADS: Hospital Anxiety and Depression Scale
IPAQ: International Physical Activity Questionnaire
MCI: Mild Cognitive Impairment
NEADL: Nottingham Extended Activities of Daily Living Scale
NHS: National Health Service
NIHR: National Institute for Health Research
NRES: National Research Ethics Service
PAL: Paired Associated Learning
PrAISED: Promoting Activity Independence and Stability in Early Dementia
RSW: Rehabilitation Support Worker
SD: Standard deviation
SHARE: Survey of Health, Ageing and Retirement in Europe
sMMSE: Standardised Mini Mental State Examination
SWM: Spatial Working Memory
TUG: Timed up and go test
